# Supplementary material for: Seed longevity is controlled by metacaspases
Source: Nat Commun. 2024 Aug 8;15:6748. doi: 10.1038/s41467-024-50848-2 (PMC11310522; doi:10.1038/s41467-024-50848-2)
Supplement: Supplementary file 1 — Supplementary Information [file 41467_2024_50848_MOESM1_ESM.pdf]

Chen Liu, Ioannis H. Hatzianestis, Thorsten Pfirrmann, Salim H. Reza, Elena A. Minina, Ali Moazzami, Simon Stael, Emilio Gutierrez–Beltran, Evgenia Pitsili, Peter Dörmann, Sabine D' Andrea, Kris Gevaert, Francisco Romero–Campero, Pingtao Ding, Moritz K. Nowack, Frank Van Breusegem, Jonathan D. G. Jones, Peter V Bozhkov, Panagiotis N. Moschou

Supplementary Figures

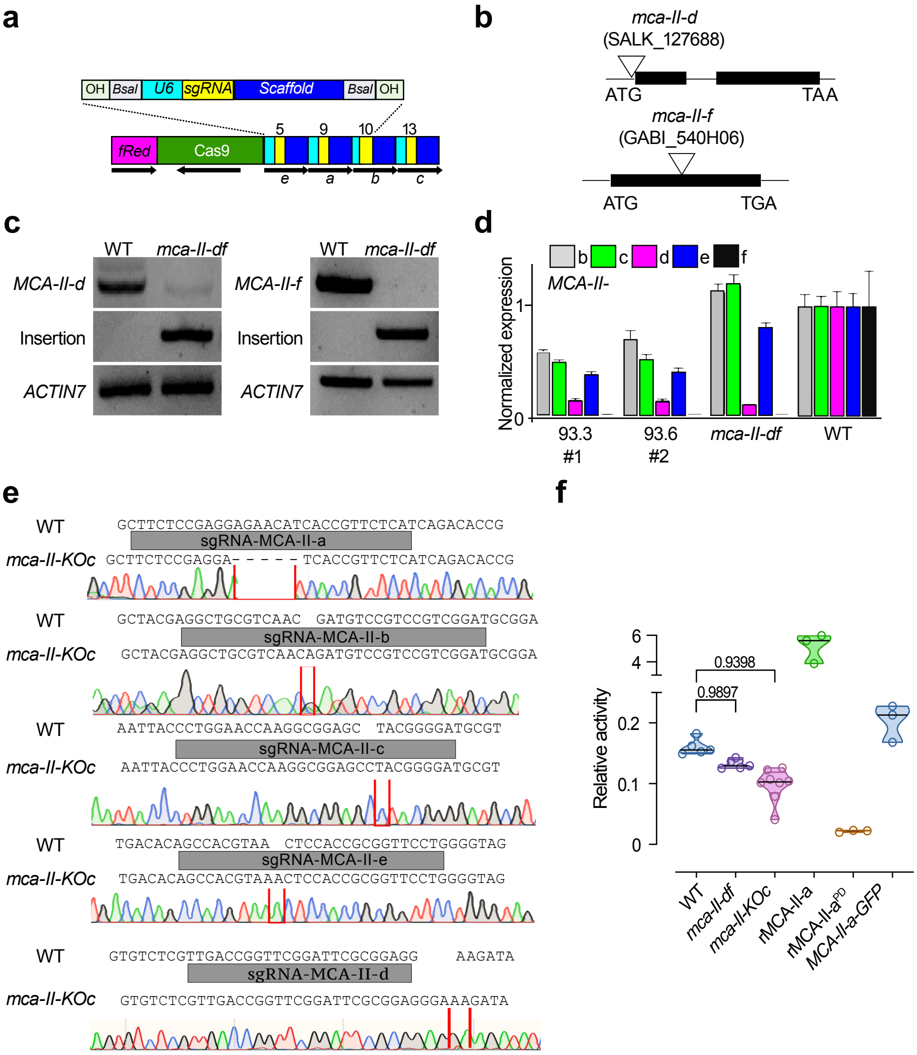

Supplementary Figure 1. Generation of a type II MCA depletion model.

(a) Schematic representation of the construct used for generating the MCA type II sextuple CRISPR mutants. The construct contained a selection marker expressed at the seed coat– (*OLE1pro:Oleosin1–fastRFP; “fRed”*), the Cas9 cassette driven by RPS5apro, and four gRNAs driven by U6. The gRNAs target the first exon of *MCA–II–a*, *–b*, *–c,* and *–e*. Note that Cas9 and gRNAs are transcribed in opposite directions, which we found to increase targeting efficiency (when compared to other constructs that we used). To obtain homozygotes, we screened >1,000 individuals, suggesting a drift from the expected Mendelian segregation. **(b)** T(transfer)–DNA insert positions (triangles) in the double mutant *mca–II–df* that was used as a background to generate the initial sextuple CRISPR mutant. ATG and TAA/TGA represent the start and the stop codons, respectively. **(c)** RT–PCR from 5–day–old seedlings showing the absence of *MCA–II–d* and *MCA–II–f* transcripts. ACTIN7 was used as a reference gene. **(d)** Relative expression of *MCA–IIs* (qRT–PCR) RNAs after normalization with WT (set at y–value “1”), in *mca–II–df*, and two individual initial sextuple *mca–II–KOc* mutant lines (#93.3 and #93.6; means ±s.d; *N* = 2 biological replicates*, n* = 3 technical replicates). **(e)** Sequencing of the gRNA targeting regions and mutation alignments for *MCA–II–a*, *–b*, *–c*, *–e*, and *–d* in the *mca–II–KOc* mutant line #93.6 which was used to build the final CRISPR line (*mca–II–KOc*). The chromatographs shown are exports from snapGene. **(f)** Quantification of the proteolytic activity on the

substrate EGR–AMC (H–Glu–Gly–Arg–7–amino–4–methylcoumarin) in WT, *mca–II–df*, and *mca–II–KOc* mutants. As controls, the recombinant purified proteins MCA–II–a (positive) and the proteolytically dead (inactive) variant MCA–II–a^{PD} (negative) were used. Recombinant proteins were purified from *Escherichia coli* as hexahistidine–tagged variants, together with total protein extract from seedlings of a line carrying the *MCA–IIapro:MCA–II–a–GFP* transgene ("MCA–II–a–GFP"). *P*–values were calculated by one–way ANOVA (N = 3*,* n = 1). Source data are provided as a Source Data file.

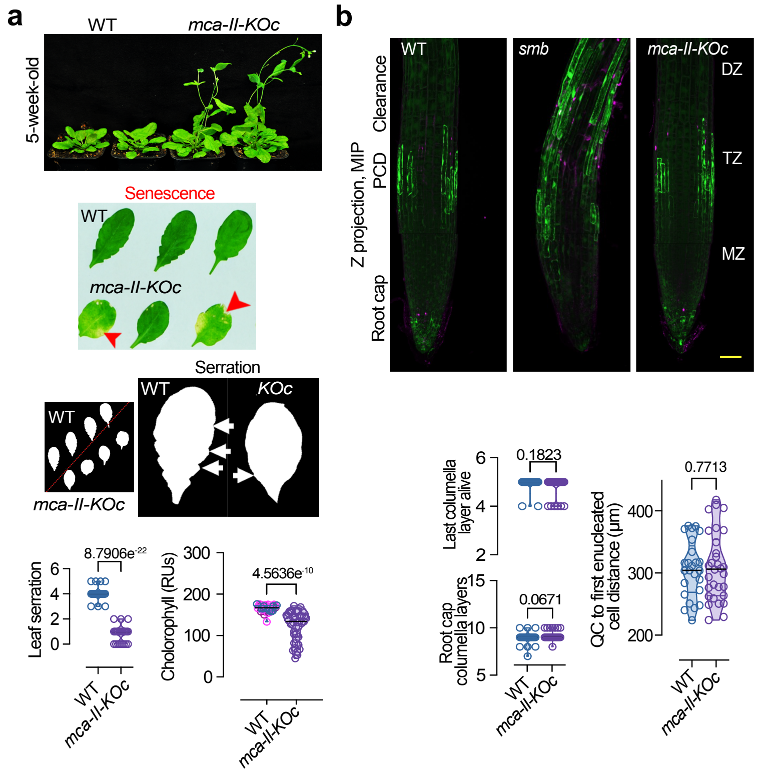

Supplementary Figure 2. Phenotypes of a type II MCA depletion model.

(a) Image of the phenotype of six–week–old (adult) WT and *mca–II–KOc* plants. Note the earlier flowering (upper panel) and senescence (lower panel) of *mca–II–KOc* (upper: short–day condition, lower: long–day condition; red arrowheads denote leaf yellowing regions indicative of senescence symptoms). Lower: image showing lack of serration (white arrows) in *mca–II–KOc* leaves, and quantification of chlorophyll from leaves of six–week–old plants and leaf serration in WT and *mca–II–KOc* plants. *P*–values were calculated by unpaired t test ($N = 3$, $n = 10$ seedlings). **(b)** Confocal micrographs from root cap PCD assay in WT, *sombrero* (*smb*; ref. [1]), and *mca–II–KOc* determined by fluorescein diacetate (FDA; green) and propidium iodide (PI; magenta). FDA stains living cells, while PI dead cells. The experiment was repeated two times ($N = 2$, $n = 20$ roots in total). The mutant *smb* shows compromised cell death in the root cap and was used as a control here. Scale bar, 20 µm. DZ, differentiation zone; TZ, transition zone; MZ, meristematic zone. Bottom: quantifications of the last columella cell alive, root cap columella cells, or the distance between the QC (quiescent center) to the first enucleated cell in the root cap in WT or *mca–II–KOc*. *P*–values were calculated by unpaired t test ($N = 3$, $n \geq 7$ roots). Source data are provided as a Source Data file.

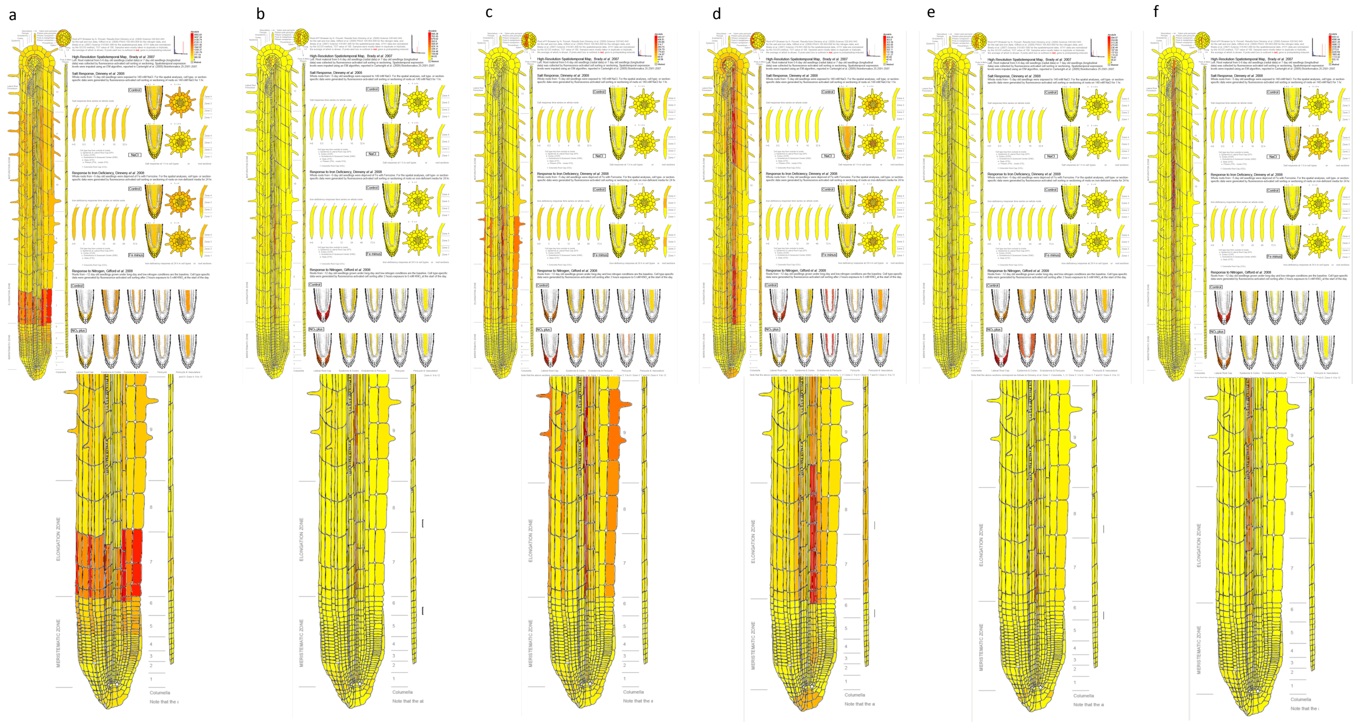

Supplementary Figure 3. MCAs type II expression patterns in primary roots.

(a)–(f) Root expression pattern of *MCA–II–a* to *MCA–II–f*. Data are from https://www.arabidopsis.org/.

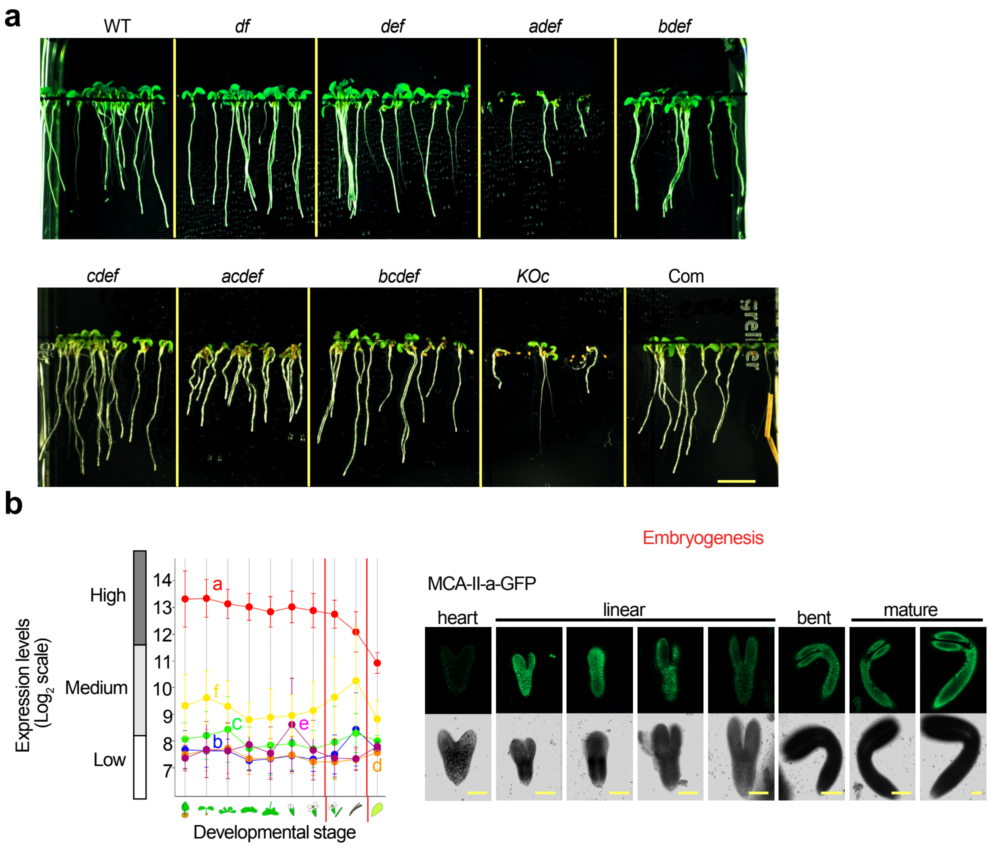

Supplementary Figure 4. Modulation of seed physiology by combinations of MCA–IIs depletions.

(a) Images from seven–day–old seedlings carrying different combinations of *MCA–II* loss–of–function mutations generated via CRISPR and a complementation line (“Com”; *PRS5apro:MCA–II–a–mNeon*) in the *mca–II–KOc* background. Note the correlation between low germination and mutations of MCA–II–a (*adef* and *acdef*). Scale bar, 1 cm. The experiment was repeated three times with similar results (*N = 3, n = 1* replicate with 10–12 seedlings). **(b)** Expression profiles (mRNA levels) of MCA–IIs at different developmental stages of embryogenesis as determined by Genevestigator (https://genevestigator.com) (left) and confocal micrographs of *MCA–II–apro:MCA–II–a–GFP* expression at different stages of embryogenesis (right). Scale bars, 7 µm.

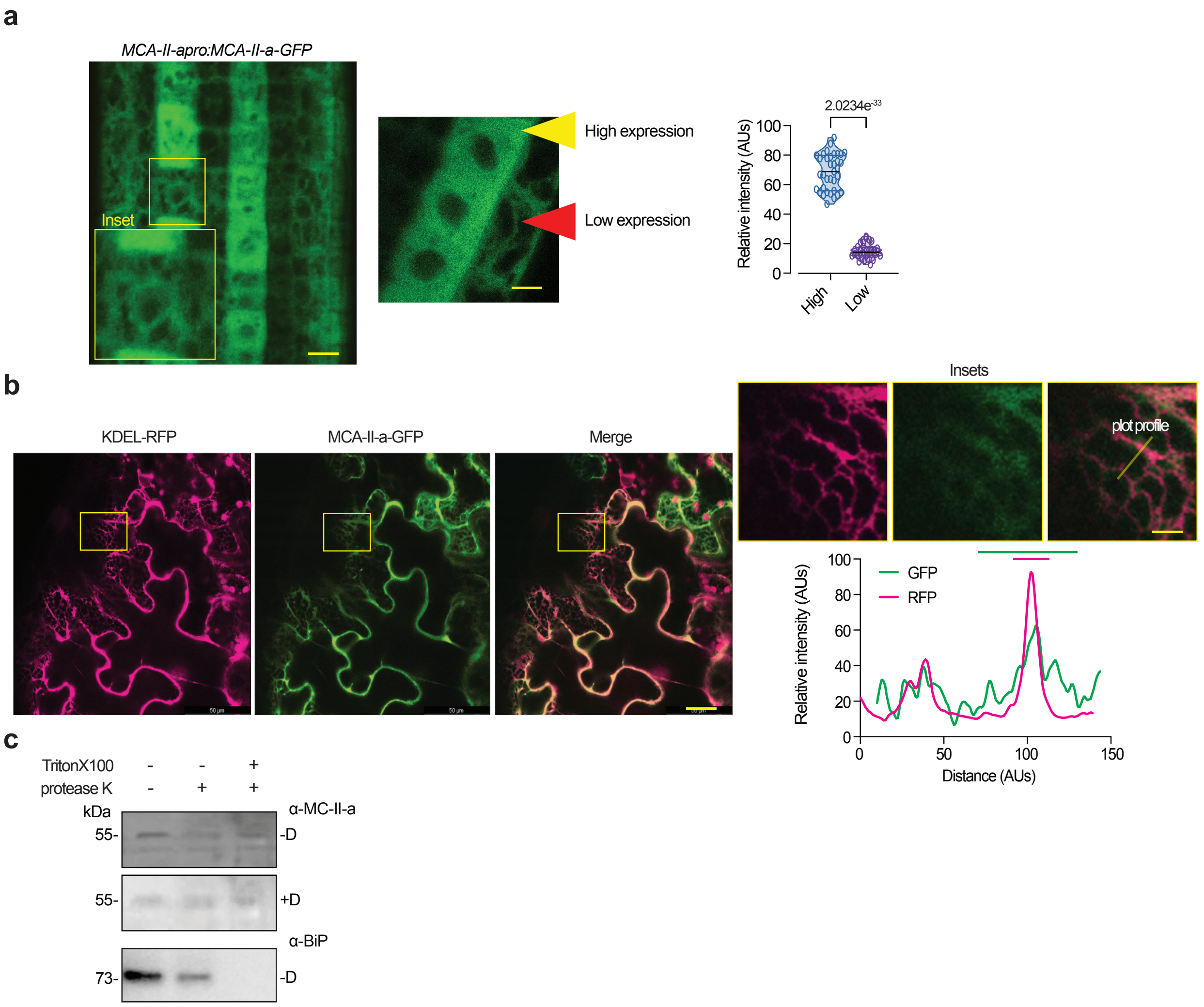

Supplementary Figure 5. Association of MCA–II–a with the ER.

(a) Confocal micrographs from root meristematic epidermal or cortex cells, showing high or low expression from lines expressing *MCA–II–apro:MCA–II–a–GFP.* Middle: detail of "high" and "low" expressing cell files. The corresponding signal quantifications are also shown (right). *P*–values were calculated by unpaired *t*–test (N = 3, n ≥ 7 roots). **(b)** Confocal micrographs from *N. benthamiana* leaf epidermal cells expressing MCA–II–a–GFP and RFP–KDEL (ER marker; both transgenes under the 35Spro). Right: insets, showing details of colocalizations and plot profile (lower). Note the spreading of the GFP signal compared to the RFP denoted by the green and magenta lines, respectively, above the major intensity peak (N = 3, n = 2 leaf spots per replicate). **(c)** Protease protection assay from microsomal fractions of WT plants. Purifications were done under two conditions, in the presence or absence of detergent (D; NP–40). Source data are provided as a Source Data file.

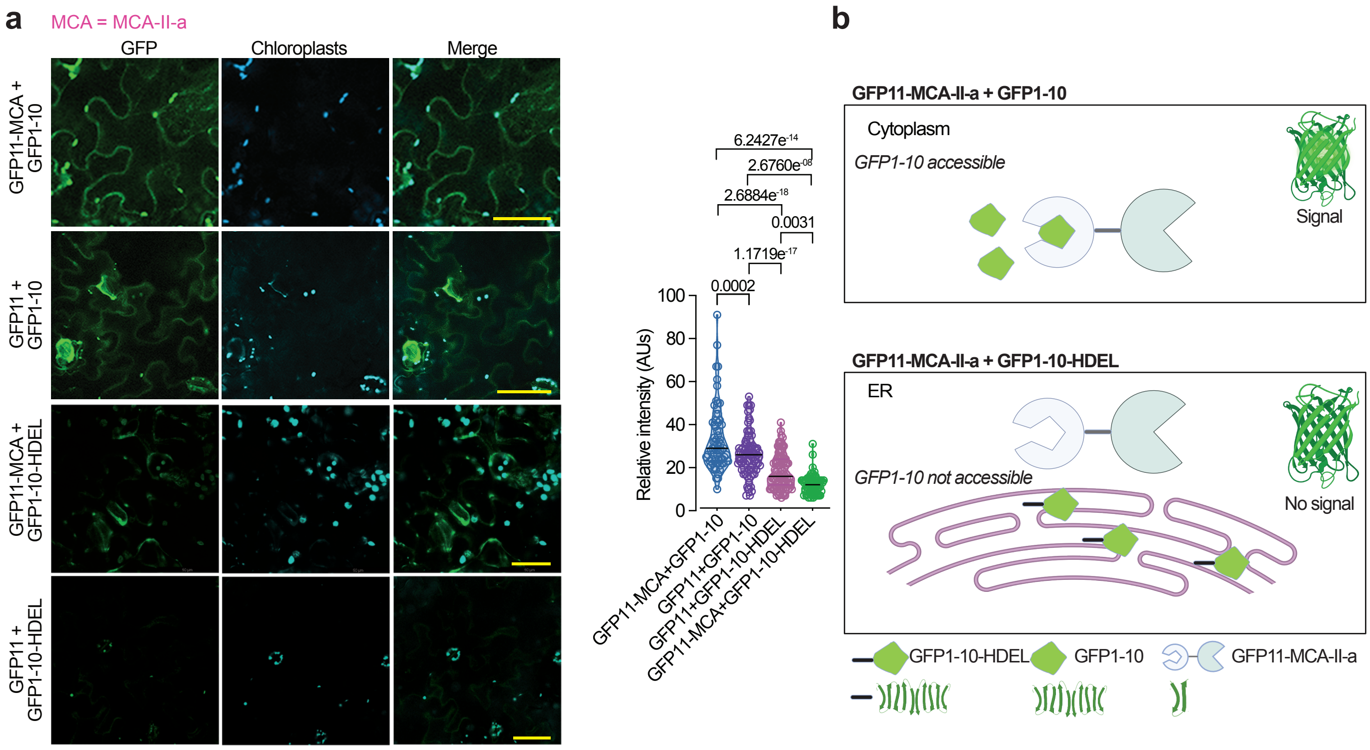

Supplementary Figure 6. Verification of MCA–II–a topology using a split–GFP detection system

(a) Confocal images of *Nicotiana benthamiana* epidermal cells co–expressing GFP1–10 (+/– HDEL) with GFP11 (+/– MCA–II–a) in different combinations under the 35Spro. This split–GFP assay is based on GFP auto–assembly when two polypeptides–the "detector" GFP1–10 (residues 1–214; fused to MCA–II–a) and the "tag" GFP11 (residues 215–230; fused to HDEL) –both non–fluorescing on their own, associate spontaneously and produce fluorescence[3]. Scale bar, 50 µm. Right: quantification of corresponding signal intensities. *P*–values were calculated by one–way ANOVA (N = 3, n = 2 leaf spots per replicate with ≥ 7 cells each). **(b)** Schematic representation of the split–GFP concept. Source data are provided as a Source Data file.

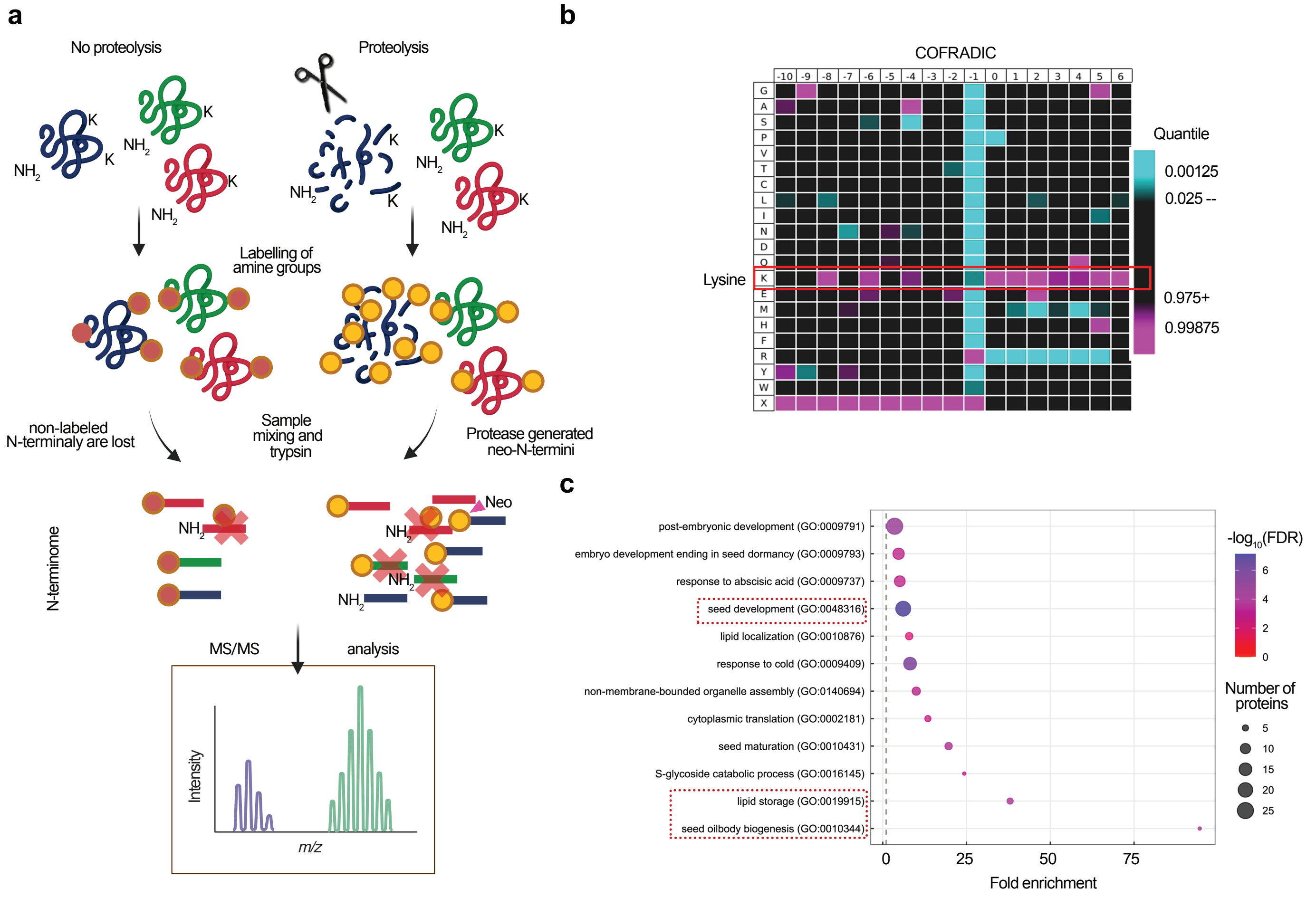

Supplementary Figure 7. Pipelines for COFRADIC analyses and corresponding gene ontologies of proteins affected in *mca–II–KOc.*

(a) Illustration of the workflow of the "N–terminomic" method known as COFRADIC (COmbined FRActional DIagonal Chromatography). This method is based on the differential labeling of amine groups of aa between the samples, followed by trypsin digestion and tandem mass spectrometry analysis, detecting the potential endogenous substrates of MCA–IIs using total seeds extract (100 mg, 3–month–old). **(b)** MCA–IIs cleavage specificity was determined using Icelogo analyses from N–termini produced from COFRADIC datasets after sequence alignment and statistical correction for Arabidopsis proteins (as in ref. [2]). The heat map shows the frequencies of amino acid residues at the P6–P10′ positions (P1 is R) and proteins rich in lysines (K) spanning the cleavage site are preferred targets of MCA–IIs at this stage (seeds). Increased or decreased amino acid residue frequencies are shown in a gradient of cyan and magenta shades. The data are from two experiments ($N = 2$, n = 1). **(c)** Gene Ontology (GO) biological term analyses of the potential endogenous substrates of MCA–IIs ($Log_2FC \geq 1$). Note the denoted by rectangular enrichment in GO terms of seed and post–embryonic development, lipid storage, and seed oil body (lipid droplet, LD) biogenesis. The S–glycoside catabolic process corresponds to BGLUs identified in the total proteome (**Fig. 2**). FDR, false discovery rate.

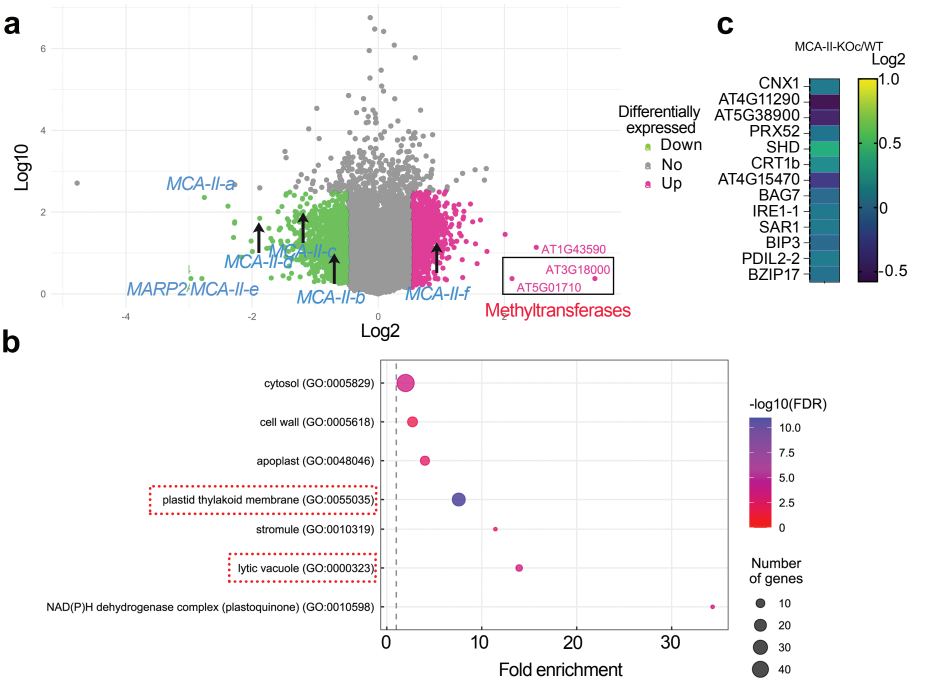

Supplementary Figure 8. RNAseq from *mca–II–KOc*, corresponding gene ontologies, and UPR–related RNA levels.

(a) Volcano plot showing differentially expressed genes (DEGs) in *mca–II–KOc* compared to the WT in the RNA–seq dataset obtained from total RNA of 3–month–old seeds ($Log_2FC \geq 0.5$ or ≤ -0.5, $N = 2$). Arrows denote the expression levels of MCA–II genes confirming the results of qRT–PCR in Supplementary Fig. 1. Note the increased expression of AT3G18000 (methyltransferase) which has been identified as critical for lipid homeostasis[4]. **(b)** Gene Ontology (GO) term analysis for Cellular Component (CC) of DEGs ($Log_2FC \geq 0.5$ or ≤ -0.5) showing a wide range of subcellular localization such as the lytic vacuole or the plastid thylakoid membrane and lumens. The enrichment of the lytic vacuole and chloroplast–associated GO terms could be justified due to enriched proteasome complex formation leading to impaired proteostasis. A characteristic example is the chloroplast regulation via the proteasome vital for the developmental transitions of plastids to chloroplasts [5]. **(c)** Heat map showing that UPR–related marker genes (selected according to ref. [6]) were not differentially expressed in *mca–II–KOc*. For example, BIP3 is a chaperone that, along with other proteins, facilitates the proper folding of newly synthesized proteins[7]. IRE1–1 is a protein kinase that is auto–activated in the presence of unfolded proteins in the ER and activates transcription factors such as bZIP60 for UPR initiation [8]. FDR, false discovery rate. Source data are provided as a Source Data file.

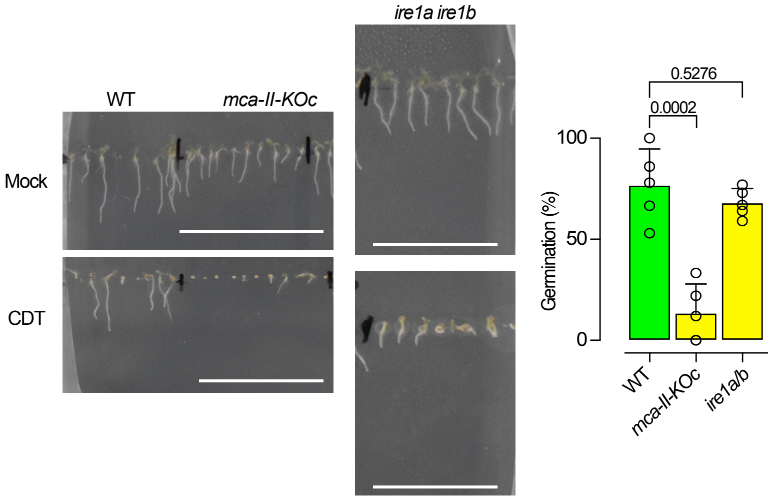

Supplementary Figure 9. Effect of controlled deterioration treatment of WT, *mca–II–KOc* and *ire1a ire1b* seeds.

Left: Images of controlled deterioration treatment (CDT) in WT, *mca–II–KOc* and *ire1a ire1b.* Scale bars, 2 cm. Right: quantification of the seed germination percentage. The experiment was repeated 3 times ($N = 3$). P–values were calculated by one–way ANOVA ($N = 2$, $n = 1$ replicate with 5 roots each). Source data are provided as a Source Data file.

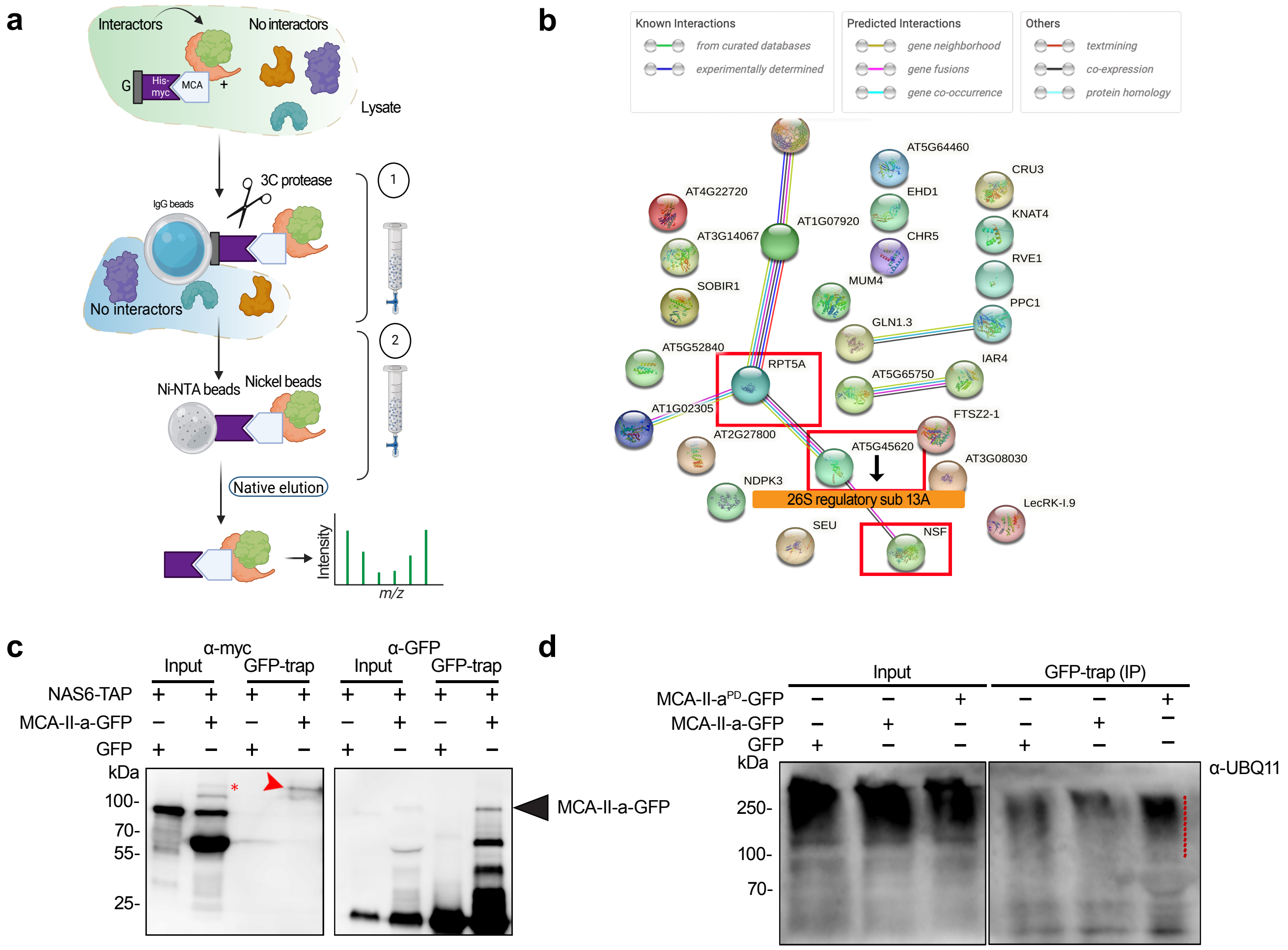

Supplementary Figure 10. Interactions of MCA–IIs with the proteasome complex.

(a) Cartoon showing the tandem affinity purification (TAP) approach with the baits *35spro:MCA–II–a–TAP*/*35spro:MCA–II–a*^{PD} (protease–dead)–TAP, and 3*5spro:MCA–II–b–TAP*/*35spro:MCA–IIb*^{PD}*–TAP* using a total extract from 7–day–old transgenic lines to investigate the potential substrates and/or interactors (details in Materials and Methods). MCA–IIa^{PD} and MCA–IIb^{PD} were used here to capture substrates, as the protease–substrate complex may be dissembled after proteolysis[9]. **(b)** Proteasomal STRING map (Search Tool for the Retrieval of Interacting Genes/Proteins) showing potential interactors obtained in TAP (red frames) and their relatively weak linkage to the proteasome. Note that the red frame marks the network enrichment of proteasome regulatory subunits for RPT5A (26S proteasome AAA–ATPase regulatory subunit particle 5b) and AT5G45620 (proteasome regulatory subunits 13A). **(c)** Immunoblot probed with α–GFP/α–myc showing the co–immunoprecipitation of MCA–II–a–GFP (bait) with NAS6–TAP–Myc from total protein extracts from *N. benthamiana* transient expression system (4–days–post–infiltration). The red asterisk and arrowhead indicate that a high molecular weight complex of NAS6 was pulled down with MCA–II–a–GFP (*N* = 2, n = 1 replicate). **(d)** Immunoblot with α–UBQ11 showing Ub–linked protein immunoprecipitation with the baits MCA–II–a–GFP or MCA–II–a^{PD}–GFP from total protein extracts from *N. benthamiana* transient expression system (4–days–post–infiltration). The dashed red line denotes the poly–Ub–linked/associated proteins in the MCA–II–a^{PD}–GFP sample (*N* = 2, n = 1 replicate).

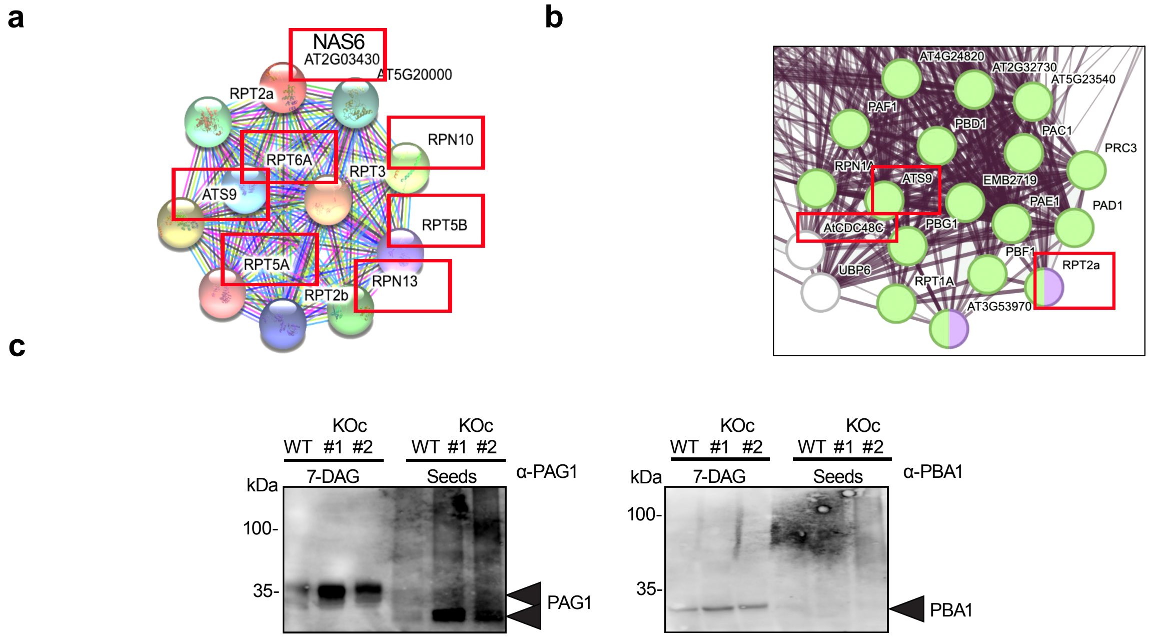

Supplementary Figure 11. Interactions of MCA–IIs with regulatory subunits of the proteasome complex.

(a) STRING map showing protein–protein interaction map of RPT5A (Regulatory Particle 5a; one of the six AAA–ATPases of the proteasome regulatory particle) and related proteasome regulatory subunits network including NAS6 (chaperon for proteasome assembly, probable 26S proteasome regulatory subunit p28) which was used in the pull–down studies in **Supplementary Figure 10d**. Note the NAS6 and CDC48 are both chaperones for proteasome assembly [10]. The ATS9 (regulatory particle non–ATPase 6, RPN6) and regulatory particle AAA–ATPase 2A (RPT2a) are shown in **c** (see below). **(b)** Part of the STRING map from the total proteome analysis (FC > 2 compared to WT) represents a cluster enriched in proteasome complex. The red arrowheads denote CDC48 (FC = 4.1, adj P = 0.67). Red cycles represent the proteasome subunits, and light blue cycles represent the regulation of proteolysis. Among those, 26S proteasome non–ATPase regulatory subunit 3 homolog A (RPN3A), 20S proteasome beta subunit D1 (PBD1) and 20S proteasome alpha subunit e1 (PAE1) were enriched 100–fold in *mca–II–KOs* mutant vs WT (three–month–old seeds, adj. P<0.001). **(c)** Immunoblots of total protein extracts probed with α–PAG1 (20S proteasome alpha subunit G–1) or α–PBA1 (20S Proteasome subunit beta 1) from *MCA–II–KOc* seedling (5 DAG) or seeds (50 seeds/sample). Note that seeds express likely a different variant of PAG1 (two gene models are predicted in arabidopsis.org). The experiment was repeated three times with similar results (N = 3, n = 1 replicates with 50 seeds' protein extract per lane).

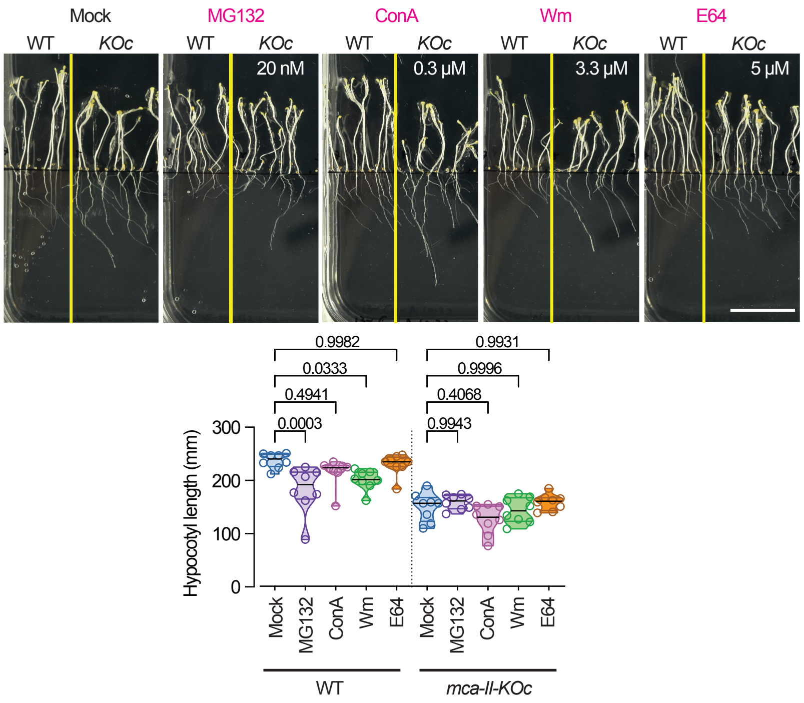

Supplementary Figure 12. Effect of proteolytic inhibitors on *mca–II–KOc* growth.

Upper panel: Image showing etiolated seedlings grown for 5 days under darkness on mock (DMSO), 20 nM MG132, 0.3 μM concanamycin (Con A; specific inhibitor of V–ATPase and thus, acidification that promotes proteolysis in the vacuole), 3.3 μM wortmannin (WM; selective inhibitor of phosphatidylinositol 3–kinase and thus, inhibitor of transport to the vacuole), and 5 μM E64D (aloxistatin; an irreversible and membrane–permeable cysteine protease inhibitor that can inhibit autophagy, which does not inhibit MCAs) containing plates. Lower panel: quantification of the corresponding hypocotyl length (units in mm). Scale bar, 20 mm. *P*–values were calculated by ordinary unpaired t–test (*N* = 2, *n* = 1 replicate with 5 hypocotyls each). Source data are provided as a Source Data file.

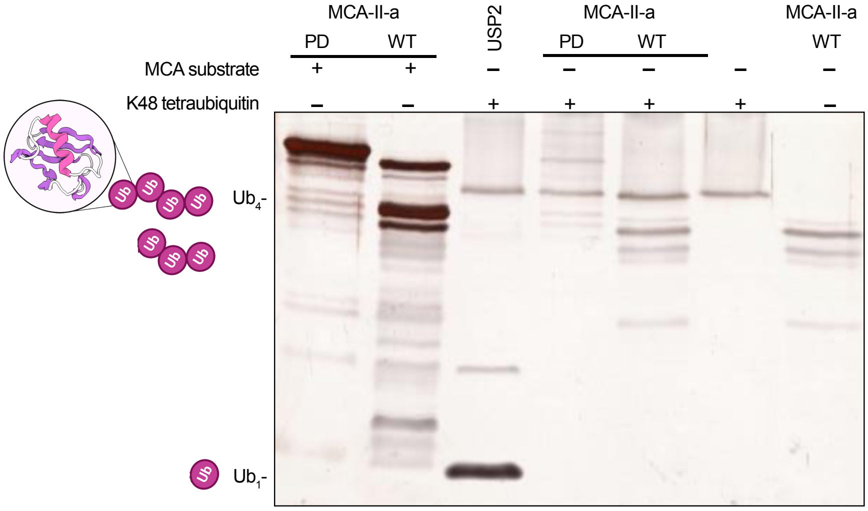

Supplementary Figure 13. Isopeptide cleavage assay for MCA–II–a.

In vitro K48 tetra–Ub isopeptide linkage type cleavage assay was conducted using the recombinant proteins of MCA–II–a and MCA–II–a^{PD} (refs. [11]). In comparison to the positive control USP2–cc[12], a deubiquitinase, which can cleave the K48 tetra–Ub isopeptide linkages, there is no obvious cleavage catalyzed by recombinant MCA–II–a. Polyacrylamide gel electrophoresis (12%) silver–stained gel. The “MCA substrate” corresponds to PROPEP1–GST, as defined in ref. [13]. The experiment was repeated two times with similar results (N = 2, n = 1 replicate with one well per sample).

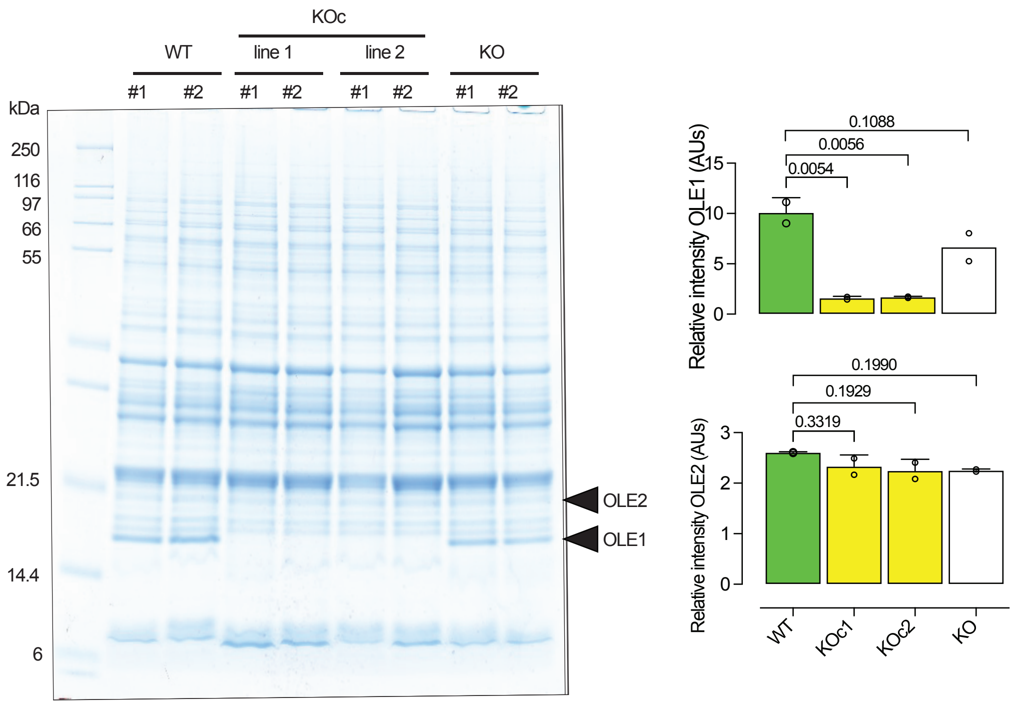

Supplementary Figure 14. Detection of oleosins accumulation by protein gels in WT and *mca–II–KOc* seeds.

Left panel: Coomassie staining of a polyacrylamide gel (12%) with seed protein extracts from WT, two individual *mca–II–KOc* lines and a non–clean *mca–II–KO* focusing on OLE1 and OLE2 (black arrowheads). Right panel: quantification of the corresponding relative band intensity compared to the band at around 21.5 kDa for the different lines. *P*–values were calculated by one–way ANOVA (*N* = 2, *n* = 2 replicates). AUs, arbitrary units. Source data are provided as a Source Data file.

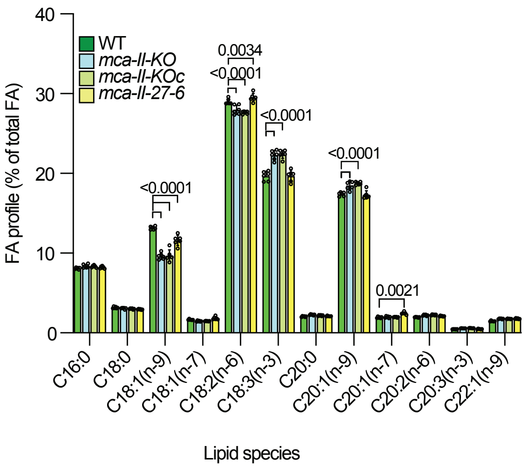

Supplementary Figure 15. Fatty acid profiling from WT, *mca–II–df*, and *mca–II–KOc*.

Fatty acid profiling of total extracts from WT, *mca–II–KO,* and *mca–II–KOc* mutant of seeds harvested at the same time (3–month–old seeds). The *mca–II–27–6* retains partial *mca–II–d* activity (see also Supplementary Fig.1 for a description). *P*–values were calculated by 2–way ANOVA; for aesthetic reasons, only those < 0.005 are shown (*N* = 6, *n* = 1 replicate with 100 mg seeds each). Source data are provided as a Source Data file.

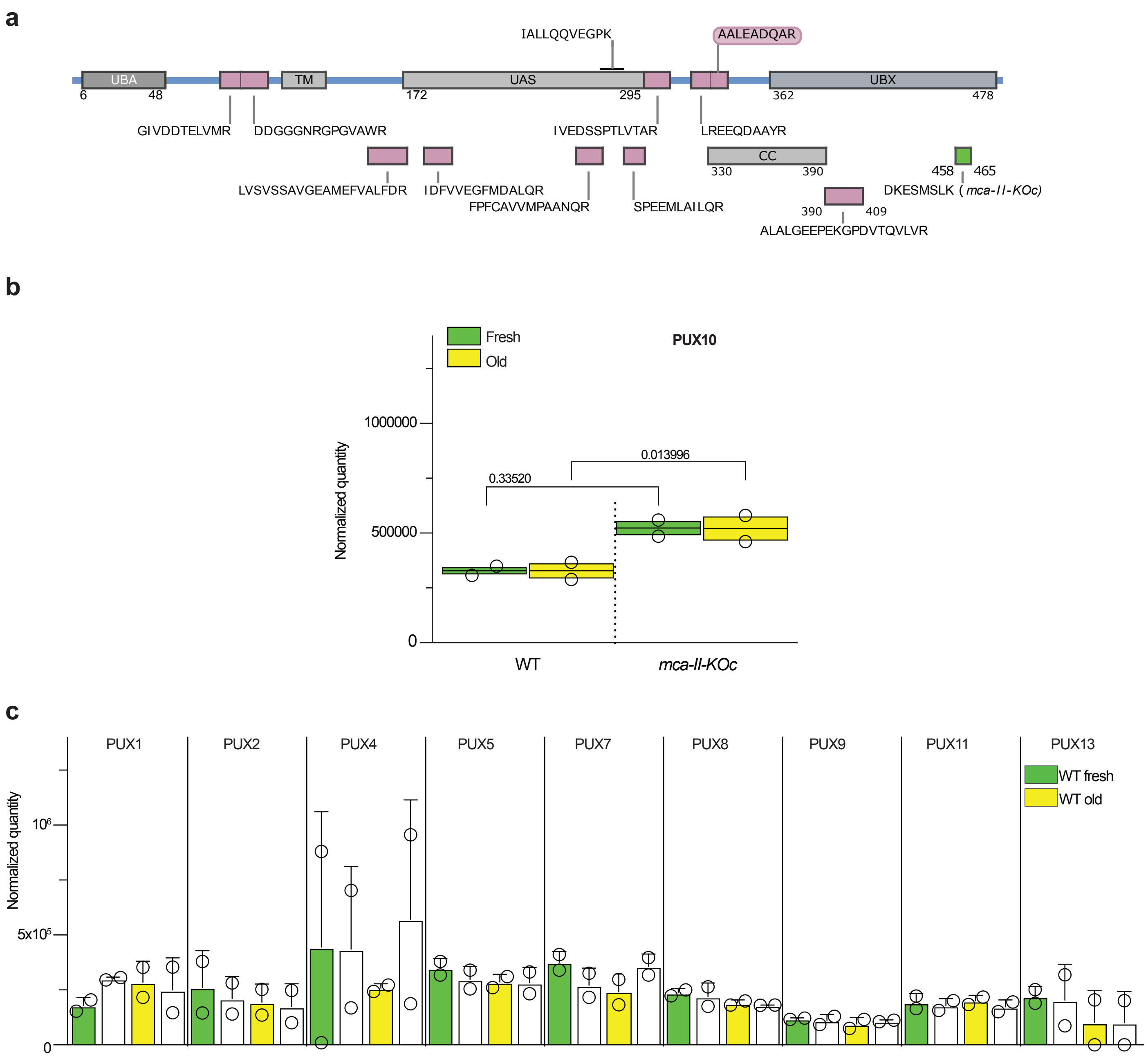

Supplementary Figure 16. Quantification of PUX proteins in WT and *mca–II–KOc* by the DIA approach.

(a) Peptides identified by DIA for the PUX10 protein in "fresh" (freshly collected) and "old" seeds (3–month–old) for the WT and *mca–II–KOc*. A peptide from the C–terminus of PUX10 (green), was only detectable in *mca–II–KOc*. **(b)** Relative quantification of PUX10 protein levels in WT and *mca–II–KOc* ("fresh" and "old" 3–month–old seeds). **(c)** Relative quantification of PUX1, 2, 4, 5, 7, 8, 9, 11, and 13 proteins (normalized to total proteome) from freshly collected ("fresh") and "old" seeds (3–month–old seeds). *P*–values were calculated by one–way ANOVA (N = 2, n = 2 replicates with 100 mg seeds for each line). Source data are provided as a Source Data file.

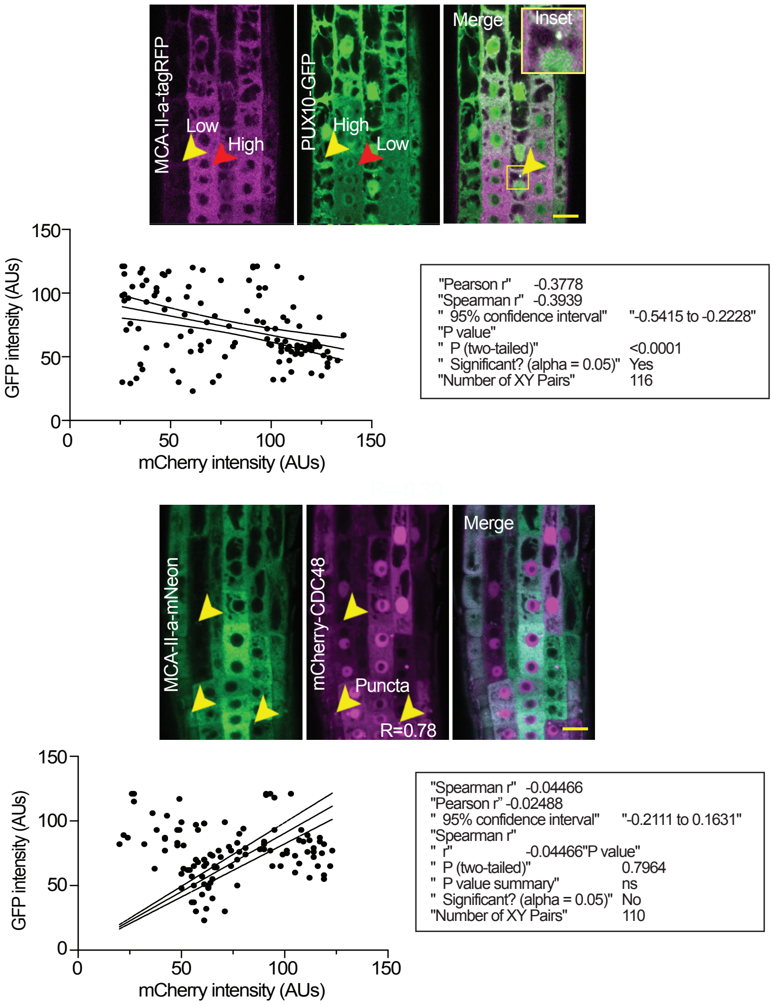

Supplementary Figure 17. Correlation between PUX10/CDC48 levels with MCA–II–a.

Upper panel: Confocal micrograph from embryonic root cells co–expressing *RPS5apro:MCA–II–a–tagRFP* and *PUX10:PUX10–GFP*. The inset in a merged micrograph shows the colocalization between PUX10 and MCA–II–a. “High” corresponds to high levels of either MCA–II–a or PUX10, while “Low” refers to reduced or non–detectable levels of the corresponding signals. Note the inverse correlation between MCA–II–a and PUX10 levels. Bottom panel: Confocal micrograph of embryonic root cells co–expressing *RPS5apro:MCA–II–a–mNeon* and *35Spro:mCherry–CDC48*. The yellow arrowheads denote structures reminiscent of lipid droplets and the highly correlated levels of CDC48 and MCA–II–a in puncta. Scale bars, 10 µm. R graphs and corresponding values denote Pearson or Spearman correlation coefficients and statistical analyses of signal intensities between tagRFP/GFP and mCherry/mNeon (a similar pipeline of statistics was used for main figures, as well). The 95% confidence intervals are shown as dashed lines. AUs, arbitrary units. Source data are provided as a Source Data file.

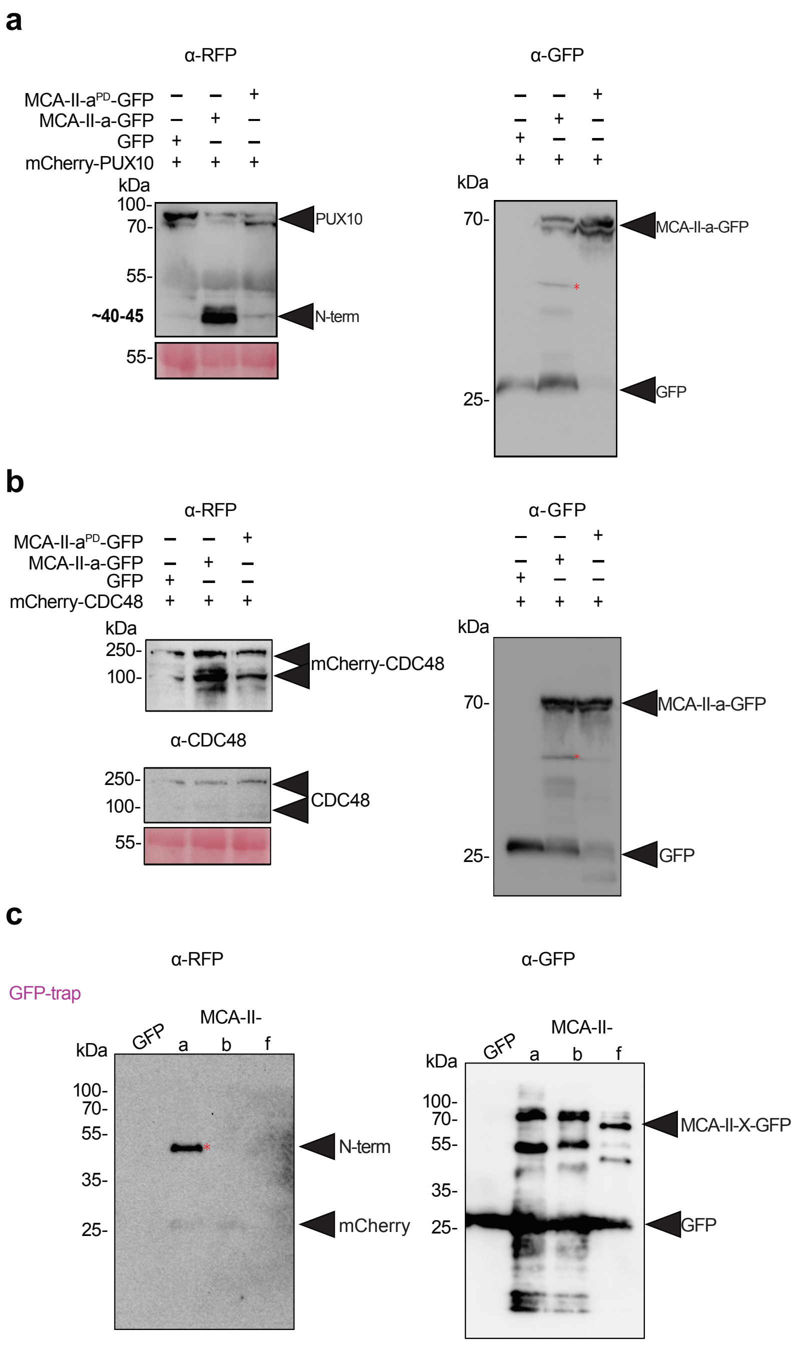

Supplementary Figure 18. MCA–II–a interaction with PUX10 and CDC48 in co–immunoprecipitation and PUX10 cleavage.

(a) and **(b)** Immunoblots probed with α–RFP (recognizing mCherry)/α–GFP/α–CDC48 detecting heterologous expressed mCherry–CDC48a, native CDC48 or mCherry–PUX10 co–expressed with MCA–II–a–GFP or the inactive variant MCA–IIa^{PD}–GFP in the *N. benthamiana* transient expression system. The proteolytic N–terminal fragment of PUX10 is also shown ("N–term"; removing the RFP, the calculated molecular weight would be around 15 kDa; *N* = 2, *n* = 2 replicates). Note that the upper bands for CDC48 (150 kDa) likely correspond to non–canonical aggregates or polymers specific for *N. benthamiana* (not observed in *A. thaliana*). The red asterisk in **(a)** indicates a product from the auto–activation of MCA–II–a (blots on the right; α–GFP). **(c)** Immunoblots showing coimmunoprecipitation between MCA–II–a–GFP, MCA–II–b–GFP, or MCA–II–f–GFP (baits) with mCherry–PUX10 in the *N. benthamiana* heterologous transient expression system (4–days post infiltration). The proteolytic fragment of PUX10 detected (N–term) by α–RFP, mainly interacting with MCA–II–a–GFP. Free GFP here was used as a negative control (*N* = 2, *n* = 2 replicates).

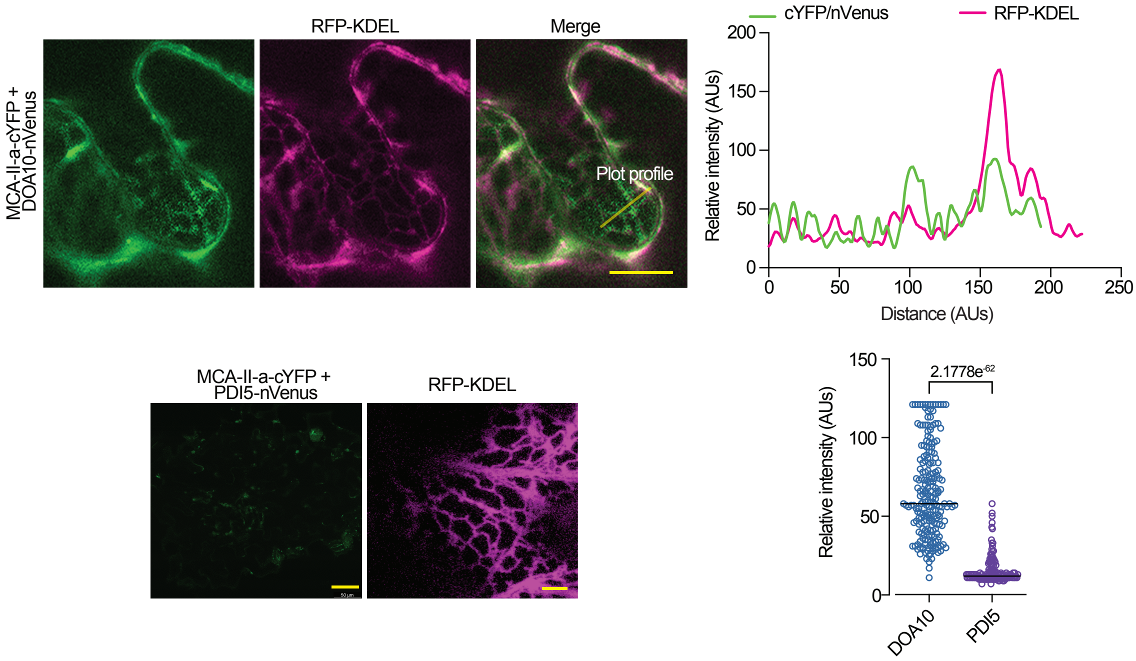

Supplementary Figure 19. MCA–II–a interaction with DOA10 in *N. benthamiana*.

Confocal images of transient co–expression of MCA–II–a–cYFP/DOA10–nVenus (BiFC assay) with RFP–KDEL (upper) or MCA–II–a–cYFP/PDI5–nVenus with RFP–KDEL (all expressed under 35Spro; lower) in *N. benthamiana* transient expression system (4–days post–infiltration; *N* = 2, n = 2 replicates). The intensity plot profile shows the spreading of the reconstituted BiFC signal versus the RFP–KDEL. Note that the DOA10/MCA–II–a BiFC signal associated with part of the ER, suggesting a specialized regulatory type. The RFP–HDEL shows the expression of the marker alone. Note that MCA–II–a did not influence ER structure. The corresponding quantifications of BiFC signals are also shown (chart). *P*–value was calculated by two-tailed Mann Whitney (*N* = 2, *n* = 2 replicates with 20 cells each). Scale bars, 2 μm (upper panel) or 50 μm/1 μm (lower panels). Source data are provided as a Source Data file.

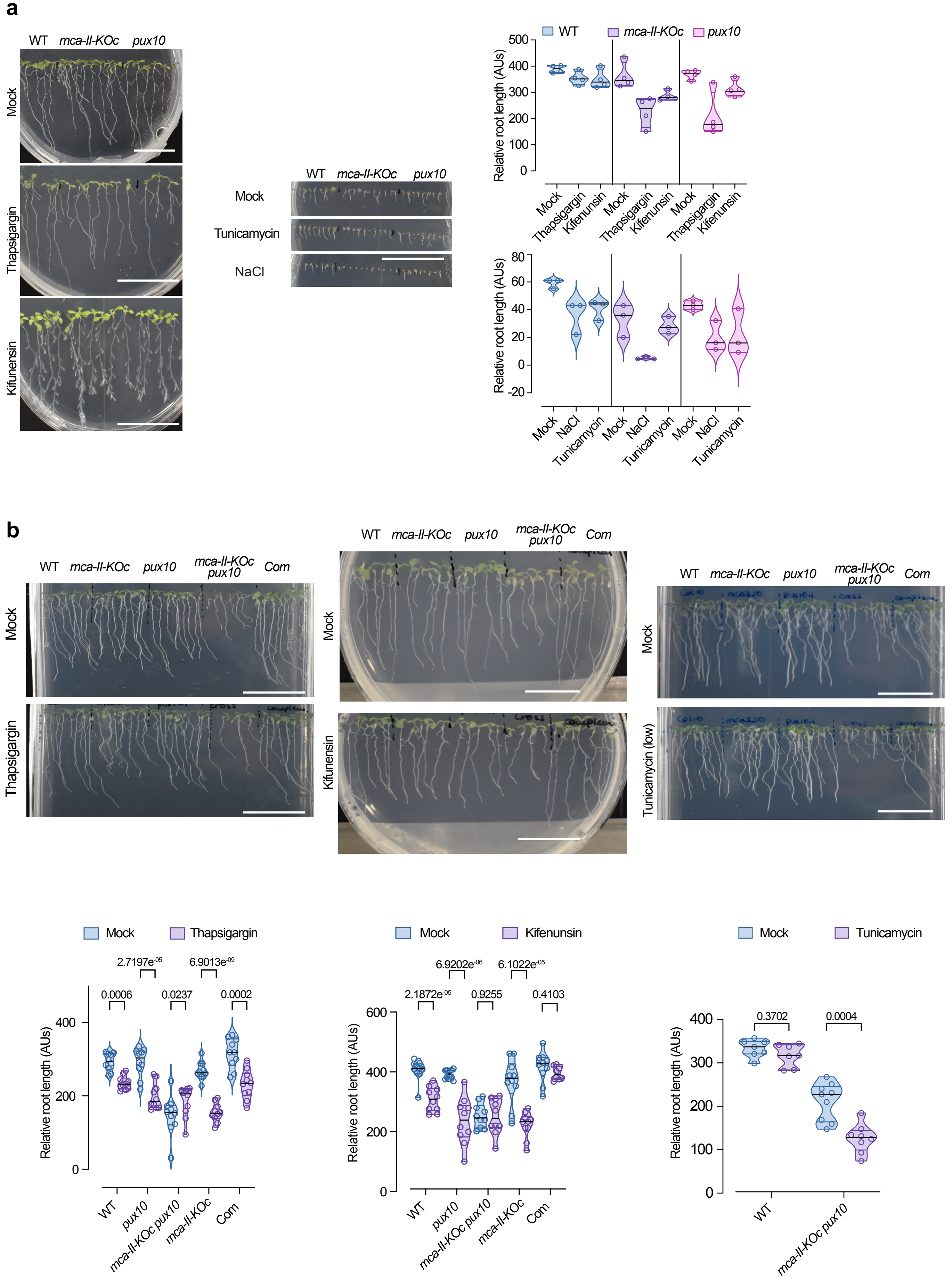

Supplementary Figure 20. Effect of ERAD–related drugs on phenotypes of WT, *mca–II–KOc,* and *pux10* mutant seedlings.

(a) Images showing the effect of treatments with thapsigargin (1 μM), kifunensin (40 μM), tunicamycin (10 nM), and NaCl on WT, *mca–II–KOc,* and *pux10* seedlings. Middle: relative quantification of root length of the above lines. *P*–values were calculated by one–way ANOVA (*N* = 2, *n* = 2 replicates). **(b)** Images showing the effect of treatments with thapsigargin (1 μM), kifunensin (40 μM), tunicamycin (1 nM; designated as "low concentration"), on WT, *pux10*, *mca–II–KOc*, and *mca–II–KOc pux10* and the Com line (*MCA–II–apro:GFP–MCA–II–a*; fresh seeds). For tunicamycin, the lower concentration is justified, due to the strong phenotype of the *mca–II–KOc pux10* in higher concentrations (almost no germination). This concentration did not affect significantly the other genotypes used. Lower panels: relative quantifications of root length of the above lines. *P*–values were calculated by 2–way ANOVA (*N* = 2, *n* = 2 replicates with 7–10 seedlings each). Source data are provided as a Source Data file.

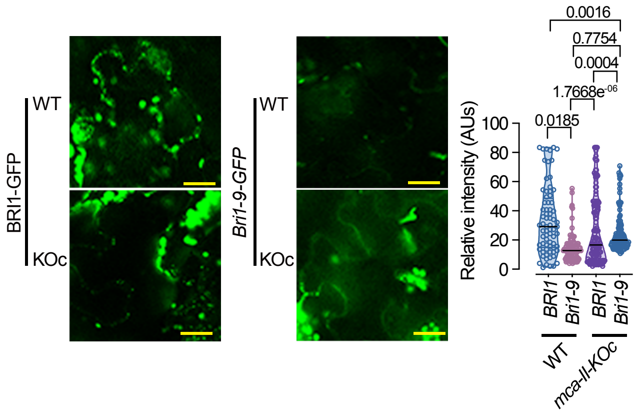

Supplementary Figure 21. Levels of BRI1 and Bri1–9 on the plasma membrane of WT or *mca–II–KOc*.

Confocal micrographs of rosette leaves from lines expressing either *RPS5apro:BRI1–GFP* or *RPS5Apro:Bri1–9–GFP* in WT or *mca–II–KOc* backgrounds. Scale bar, 5 µm. Right: corresponding quantification of the relative intensity compared to the cytoplasm of *BRI1–GFP* and *Bri1–9–GFP* proteins in WT and *mca–II–KOc* is also shown. *P*–values were calculated by one–way ANOVA (*N* = 2, *n* = 3 replicates with multiple cells). Source data are provided as a Source Data file.

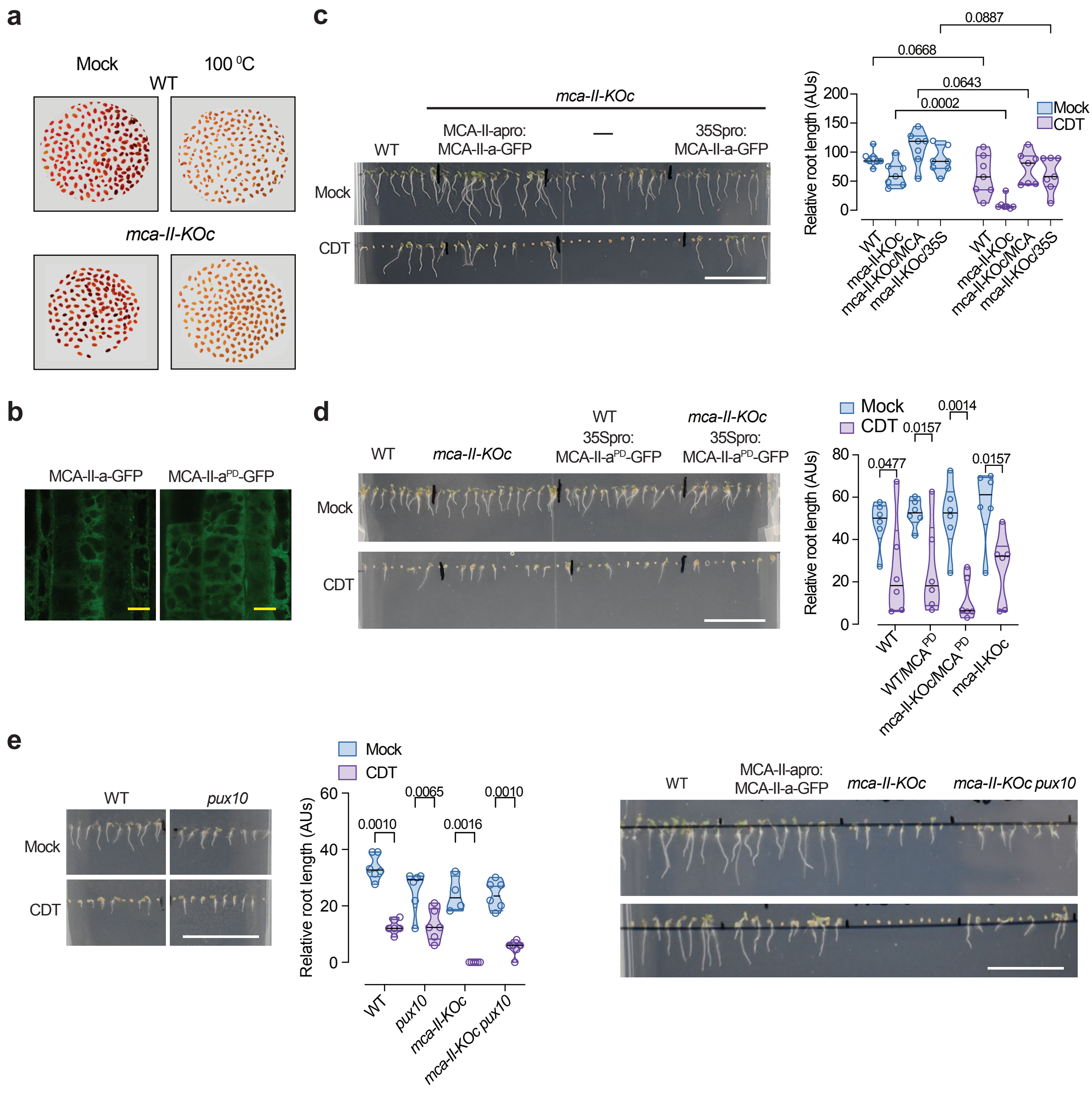

Supplementary Figure 22. Effect of CDT on seed germination of *mca–II–KOc* complemented lines and *mca–II–KOc pux10*.

(a) Seed viability tests with tetrazolium red staining in freshly harvested seeds of WT and *mca–II–KOc*. Seeds treated at 100ºC represent a positive control for dead seeds (*N* = 3, n = 1 replicate). **(b)** Confocal images of embryonic epidermal root cells from seedlings expressing either MCA–II–a–GFP or its proteolytic–dead mutated version MCA–II–a^{PD}–GFP. Scale bar, 10 µm (*N* = 2, n = 3 roots, 5 DAG). **(c)** Left: CDT in WT, *MCA–II–apro:MCA–II–a–GFP/mca–II–KOc, mca–II–KOc and 35Spro:MCA–II–a–GFP/ mca–II–KOc* seeds. The experiment was repeated 3 times with similar results (*N* = 3). Right panel: quantification of root length from the germinated seeds in both Mock and CDT conditions. *P*–value is calculated by one–way ANOVA (*N* = 3, n= 2 replicates with 5 roots each). **(d)** Left: CDT in WT, *35Spro:MCA–II–a–GFP^{PD}/mca–II–KOc,* and *35Spro:MCA–II–a–GFP^{PD}/WT* seeds. The experiment was repeated 3 times with similar results (*N* = 3). Right panel: quantification of root length from the germinated seeds in both Mock and CDT conditions. *P*–value is calculated by one–way ANOVA (*N* = 3, n= 2 replicates with 5 roots each). **(e)** Left: CDT in WT, and *pux10* seeds. Right: CDT in WT, and *mca–II–KOc pux10* seeds. The experiment was repeated 3 times with similar results (*N* = 3). Right panel: quantification of root length from the germinated seeds in both Mock and CDT conditions. *P*–value is calculated by one–way ANOVA (*N* = 3, n= 2 replicates with 5 roots each). The quantification of root length from the germinated seeds in both Mock and CDT conditions is also shown. *P*–values were calculated by two–way ANOVA (*N* = 3, n= 2 replicates with 5 roots each). Source data are provided as a Source Data file.

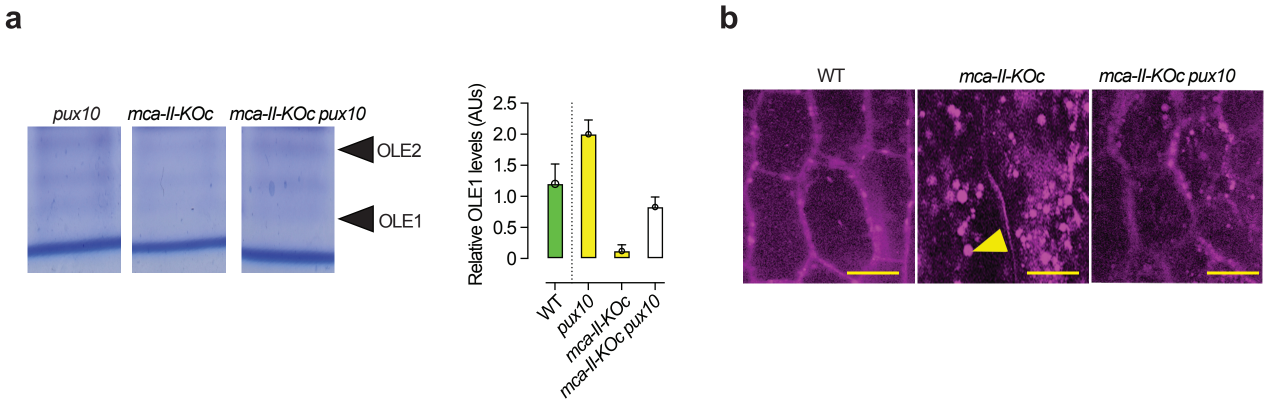

Supplementary Figure 23. Levels of OLE1, OLE2 in *mca–II–KOc* and *mca–II–KOc pux10* and corresponding oil droplet dynamics.

(a) Coomassie staining of a polyacrylamide gel (12%) with seed protein extracts from WT, *mca–II–KOc*, and *mca–II–KOc pux10* focusing on OLE1 and OLE2 (black arrowheads). Right panel: quantification of the corresponding relative band intensity compared to the band at around 21.5 kDa for the different lines. AUs, arbitrary units. **(b)** Confocal micrographs from WT, *mca–II–KOc*, and *mca–II–KOc pux10* counterstained with LipidTOX in the embryonic hypocotyl regions after a two–day stratification. An example of lipid droplets is denoted with the yellow arrowhead. Scale bars, 15 µm. Source data are provided as a Source Data file.

Supplementary References

1. Fendrych M*, et al.* Programmed cell death controlled by ANAC033/SOMBRERO determines root cap organ size in Arabidopsis. *Curr Biol* **24**, 931-940 (2014).

2. Colaert N, Helsens K, Martens L, Vandekerckhove J, Gevaert K. Improved visualization of protein consensus sequences by iceLogo. *Nature methods* **6**, 786-787 (2009).

3. Xie W, Nielsen ME, Pedersen C, Thordal-Christensen H. A Split-GFP Gateway Cloning System for Topology Analyses of Membrane Proteins in Plants. *Plos One* **12**, e0170118 (2017).

4. Chen W*, et al.* NMT1 and NMT3 N-Methyltransferase Activity Is Critical to Lipid Homeostasis, Morphogenesis, and Reproduction. *Plant Physiol* **177**, 1605-1628 (2018).

5. Ling Q*, et al.* Ubiquitin-dependent chloroplast-associated protein degradation in plants. *Science* **363**, eaav4467 (2019).

6. Srivastava R*, et al.* Response to Persistent ER Stress in Plants: A Multiphasic Process That Transitions Cells from Prosurvival Activities to Cell Death. *The Plant Cell* **30**, 1220-1242 (2018).

7. Maruyama D, Sugiyama T, Endo T, Nishikawa S-i. Multiple BiP Genes of Arabidopsis thaliana are Required for Male Gametogenesis and Pollen Competitiveness. *Plant Cell Physiol* **55**, 801-810 (2014).

8. Deng Y, Humbert S, Liu J-X, Srivastava R, Rothstein SJ, Howell SH. Heat induces the splicing by IRE1 of a mRNA encoding a transcription factor involved in the unfolded protein response in Arabidopsis. *Proceedings of the National Academy of Sciences* **108**, 7247-7252 (2011).

9. Rei Liao J-Y, van Wijk KJ. Discovery of AAA+ Protease Substrates through Trapping Approaches. *Trends Biochem Sci* **44**, 528-545 (2019).

10. Rosnoblet C*, et al.* The chaperone-like protein Cdc48 regulates ubiquitin-proteasome system in plants. *Plant, Cell & Environment* **44**, 2636-2655 (2021).

11. Tsiatsiani L*, et al.* The Arabidopsis metacaspase9 degradome. *Plant Cell* **25**, 2831-2847 (2013).

12. Catanzariti AM, Soboleva TA, Jans DA, Board PG, Baker RT. An efficient system for high-level expression and easy purification of authentic recombinant proteins. *Protein Sci* **13**, 1331-1339 (2004).

13. Hander T*, et al.* Damage on plants activates Ca(2+)-dependent metacaspases for release of immunomodulatory peptides. *Science* **363**, (2019).
