# Supplementary material for: Seed longevity is controlled by metacaspases
Source: Nat Commun. 2024 Aug 8;15:6748. doi: 10.1038/s41467-024-50848-2 (PMC11310522; doi:10.1038/s41467-024-50848-2)
Supplement: Supplementary file 3 — Description of additional supplementary files [file 41467_2024_50848_MOESM3_ESM.pdf]

Description of Additional Supplementary Files

Supplementary Data 1: Nomenclature of metacaspases used in this study.

Supplementary Data 2: MCA-I and MCA-II detected from DIA (data extracted from Supplementary File 1 in https://zenodo.org/records/12684164).

Supplementary Data 3: Proteomics of WT and mca-IIKOc seeds. Proteins with an abundant ratio ≥ 2 were considered enriched.

Supplementary Data 4: N-terminomics dataset from WT and mca-II-KOc 3-days-old seedlings.

Supplementary Data 5: RNAseq Dataset from WT and mca-II-KOc 3-day-old seedlings. DEGs were considered those with log2FC ≥ 0.5 or ≤ -0.5.

Supplementary Data 6: Analysis of identified proteins from DIA-derived proteomic dataset (Supplemental Data 1) focusing on genes implicated in RIDD, ERAD, and UPR pathways.

Supplementary Data 7: Interactome Dataset of MCA-IIa and b as well as their corresponding inactive Proteolytically-Dead variants (MCA-II-a/bPD, with catalytic Cys replaced with Ala). These proteins were used as baits for affinity purification followed by LCMS/MS.

Supplementary Data 8: Analysis of identified Oleosins, Caleosins, and Steroleosins from DIA-derived proteomic dataset (in https://zenodo.org/records/12684164). Significant changes were considered for those with log2FC ≥ 0.5 or ≤ -0.5.

Supplementary Data 9: Lipidomic analyses (determination of FA content) in seeds of WT and 3 individual mca-II-KO mutant lines.

Supplementary Data 10: Quantification of PUX peptides from DIA derived proteomic dataset (https://zenodo.org/records/12684164).

Supplementary Table 11: Protein sequences of metacaspases used in the study.

Supplementary Data 12: Primers used in this study.
