# Supplementary material for: Seed longevity is controlled by metacaspases
Source: Nat Commun. 2024 Aug 8;15:6748. doi: 10.1038/s41467-024-50848-2 (PMC11310522; doi:10.1038/s41467-024-50848-2)
Supplement: Supplementary file 16 — Reporting Summary [file 41467_2024_50848_MOESM16_ESM.pdf]

nature portfolio

Corresponding author(s): Panagiotis Moschou

Last updated by author(s): Jul 7, 2024

Reporting Summary

Nature Portfolio wishes to improve the reproducibility of the work that we publish. This form provides structure for consistency and transparency in reporting. For further information on Nature Portfolio policies, see our Editorial Policies and the Editorial Policy Checklist.

Statistics

For all statistical analyses, confirm that the following items are present in the figure legend, table legend, main text, or Methods section.

n/a	Confirmed	
☐	☒	The exact sample size (*n*) for each experimental group/condition, given as a discrete number and unit of measurement
☐	☒	A statement on whether measurements were taken from distinct samples or whether the same sample was measured repeatedly
☐	☒	The statistical test(s) used AND whether they are one- or two-sided<br>*Only common tests should be described solely by name; describe more complex techniques in the Methods section.*
☐	☒	A description of all covariates tested
☐	☒	A description of any assumptions or corrections, such as tests of normality and adjustment for multiple comparisons
☐	☒	A full description of the statistical parameters including central tendency (e.g. means) or other basic estimates (e.g. regression coefficient) AND variation (e.g. standard deviation) or associated estimates of uncertainty (e.g. confidence intervals)
☐	☒	For null hypothesis testing, the test statistic (e.g. *F*, *t*, *r*) with confidence intervals, effect sizes, degrees of freedom and *P* value noted<br>*Give P values as exact values whenever suitable.*
☒	☐	For Bayesian analysis, information on the choice of priors and Markov chain Monte Carlo settings
☒	☐	For hierarchical and complex designs, identification of the appropriate level for tests and full reporting of outcomes
☐	☒	Estimates of effect sizes (e.g. Cohen's *d*, Pearson's *r*), indicating how they were calculated

Our web collection on statistics for biologists contains articles on many of the points above.

Software and code

Policy information about availability of computer code

Data collection	Data were plotted in EXCEL and transferred to GraphPad Prism v. 10.2.0.
Data analysis	Graphs were generated by GraphPad Prism v. 10.2.0. Pearson or Spearman correlation coefficients on images were calculated via Fiji, coloc2 tool or manually and plotted in GraphPad Prism. Exact P-values <0.0001 were calculated with EXCEL 365 using ANOVA.

For manuscripts utilizing custom algorithms or software that are central to the research but not yet described in published literature, software must be made available to editors and reviewers. We strongly encourage code deposition in a community repository (e.g. GitHub). See the Nature Portfolio guidelines for submitting code & software for further information.

Data

Policy information about availability of data

All manuscripts must include a data availability statement. This statement should provide the following information, where applicable:

- Accession codes, unique identifiers, or web links for publicly available datasets
- A description of any restrictions on data availability
- For clinical datasets or third party data, please ensure that the statement adheres to our policy

All data, code, and materials used in the analysis are available and were deposited in public repositories. The data are available from the corresponding author with detailed explanations upon reasonable request. Source data are provided as a Source Data file along with this paper. Gene sequence information of MCAs is provided in Supplementary Tables 1 and 4. The mass spectrometry proteomics data have been deposited to the ProteomeXchange Consortium via the PRIDE

partner repository with the dataset identifiers PXD048890 and PXD049287. The raw RNAseq data of this article have been deposited to the BioStudies database with the dataset accession number S-BSST1310.

Research involving human participants, their data, or biological material

Policy information about studies with human participants or human data. See also policy information about sex, gender (identity/presentation), and sexual orientation and race, ethnicity and racism.

Reporting on sex and gender	Not applicable
Reporting on race, ethnicity, or other socially relevant groupings	Not applicable
Population characteristics	Not applicable
Recruitment	Not applicable
Ethics oversight	Biological use of materials have been accepted by the European Union (Ethics Commitee), the University of Crete and the Swedish University of Agricultural Sciences. These are the institutes were the handling and use of materials was done.

Note that full information on the approval of the study protocol must also be provided in the manuscript.

Field-specific reporting

Please select the one below that is the best fit for your research. If you are not sure, read the appropriate sections before making your selection.

☒ Life sciences ☐ Behavioural & social sciences ☐ Ecological, evolutionary & environmental sciences

For a reference copy of the document with all sections, see nature.com/documents/nr-reporting-summary-flat.pdf

Life sciences study design

All studies must disclose on these points even when the disclosure is negative.

Sample size	Plant materials used are stated in the corresponding legends and in methods. The sample size used is described at the figure legends and N corresponds to biological replicates, while "n"denotes the population size.
Data exclusions	Exclusion of outliers was done in the cases of saturated pixel intensity in image analytics.
Replication	Two independent replicates were used for RNAseq analyses and proteomics, while for all other analyses three biological independent replicates were done, unless otherwise stated (N>3).
Randomization	Double-blind experimentation was done in the cases where random genotypes were used, collected and analyzed.
Blinding	Randomization was used in the case of bri1-9 complementation phenotypes.

Reporting for specific materials, systems and methods

We require information from authors about some types of materials, experimental systems and methods used in many studies. Here, indicate whether each material, system or method listed is relevant to your study. If you are not sure if a list item applies to your research, read the appropriate section before selecting a response.

Materials & experimental systems

n/a	Involved in the study	
☐	☒	Antibodies
☒	☐	Eukaryotic cell lines
☒	☐	Palaeontology and archaeology
☒	☐	Animals and other organisms
☒	☐	Clinical data
☒	☐	Dual use research of concern
☐	☒	Plants

Methods

n/a	Involved in the study	
☒	☐	ChIP-seq
☒	☐	Flow cytometry
☒	☐	MRI-based neuroimaging

Antibodies

Antibodies used	All the antibodies used are described in methods. anti-MCA-II-a was customly produced. Others are against: VCP1, OLE1, GFP, RFP, UBQ11, BIP, ACTIN, PBA, PAG
Validation	anti-MCA-II-a was validated using mca-II-a mutants (Fig. 1).

Dual use research of concern

Policy information about dual use research of concern

Hazards

Could the accidental, deliberate or reckless misuse of agents or technologies generated in the work, or the application of information presented in the manuscript, pose a threat to:

No	Yes	
☒	☐	Public health
☒	☐	National security
☒	☐	Crops and/or livestock
☒	☐	Ecosystems
☒	☐	Any other significant area

Experiments of concern

Does the work involve any of these experiments of concern:

No	Yes	
☒	☐	Demonstrate how to render a vaccine ineffective
☒	☐	Confer resistance to therapeutically useful antibiotics or antiviral agents
☒	☐	Enhance the virulence of a pathogen or render a nonpathogen virulent
☒	☐	Increase transmissibility of a pathogen
☒	☐	Alter the host range of a pathogen
☒	☐	Enable evasion of diagnostic/detection modalities
☒	☐	Enable the weaponization of a biological agent or toxin
☒	☐	Any other potentially harmful combination of experiments and agents

Plants

Seed stocks	Columbia-0 (Col-0) ecotype was used unless stated otherwise. The following mutants were used smbSO, puxl0-1 (SAIL_1187), mca-II-d (SALK_127688), and mca-11-f (GAB1_540H06). The bril-9 mutant used in this study was in Ws genetic background.
Novel plant genotypes	Trangenic plants were generated in this study. The editor used is Cas9 and various gRNAs were used. Transgenic lines are listed in the paper. The plCSL binary vector series was utilized to generate CRISPR lines. Primer sequences used for the amplification of promoters and genes are listed in Table 5.
Authentication	The generated CRISPR lines described were back-crossed to wild-type Col-0 to reduce any unwanted and off-target effects and two independent lines were confirmed, carrying the same set of allelic mutations. Bi-allelic plants that were generated were not considered further and were discarded from analysis of the main findings of the paper.

April 2023
